# Integrin-α5 Coordinates Assembly of Posterior Cranial Placodes in Zebrafish and Enhances Fgf-Dependent Regulation of Otic/Epibranchial Cells

**DOI:** 10.1371/journal.pone.0027778

**Published:** 2011-12-02

**Authors:** Neha Bhat, Bruce B. Riley

**Affiliations:** Department of Biology, Texas A&M University, College Station, Texas, United States of America; National University of Singapore, Singapore

## Abstract

Vertebrate sensory organs develop in part from cranial placodes, a series of ectodermal thickenings that coalesce from a common domain of preplacodal ectoderm. Mechanisms coordinating morphogenesis and differentiation of discrete placodes are still poorly understood. We have investigated whether placodal assembly in zebrafish requires Integrin- α5 (*itga5*), an extracellular matrix receptor initially expressed throughout the preplacodal ectoderm. Morpholino knockdown of *itga*5 had no detectable effect on anterior placodes (pituitary, nasal and lens), but posterior placodes developed abnormally, resulting in disorganization of trigeminal and epibranchial ganglia and reduction of the otic vesicle. Cell motion analysis in GFP-transgenic embryos showed that cell migration in *itga5* morphants was highly erratic and unfocused, impairing convergence and blocking successive recruitment of new cells into these placodes. Further studies revealed genetic interactions between *itga5* and Fgf signaling. First, *itga5* morphants showed changes in gene expression mimicking modest reduction in Fgf signaling. Second, *itga*5 morphants showed elevated apoptosis in the otic/epibranchial domain, which was rescued by misexpression of Fgf8. Third, knockdown of the Fgf effector *erm* had no effect by itself but strongly enhanced defects in *itga5* morphants. Finally, proper regulation of *itga5* requires *dlx3b/4b* and *pax8*, which are themselves regulated by Fgf. These findings support a model in which *itga5* coordinates cell migration into posterior placodes and augments Fgf signaling required for patterning of these tissues and cell survival in otic/epibranchial placodes.

## Introduction

Development of cranial sensory organs in vertebrates requires essential contributions from transient embryonic structures termed cranial placodes. Cranial placodes form during early segmentation stages as a series of epithelial thickenings adjacent to developing brain tissue [1], [2]. The anterior-most placodes produce the anterior pituitary, olfactory epithelium, and the lens of the eye. Amongst more posterior placodes, the otic placode produces the entire inner ear, including the complex epithelial labyrinth, internal sensory epithelia, and all of its innervating neurons; and trigeminal and epibranchial placodes produce a segmental array of sensory ganglia that innervate much of the craniofacial and pharyngeal apparatus. Despite their morphological and functional diversity, all cranial placodes arise from a common domain of preplacodal ectoderm that forms earlier around the anterior neural plate [2], [3]. Specification of preplacodal ectoderm involves a sequence of signaling interactions that occur during blastula and gastrula stages, culminating in expression of a characteristic set of transcription factor genes near the end of gastrulation [4]–[8]. This contiguous domain of gene expression subsequently breaks into discrete clusters of cells that generate the various diverse placodes.

Lineage studies in zebrafish and chick indicate that resolution of preplacodal ectoderm into discrete placodes requires active cell migration and rearrangement. For example, precursors of the anterior pituitary, olfactory and lens placodes are initially intermixed but subsequently sort out to form their respective placodes [9]–[12]. In the case of the olfactory placode, precursors converge into a compact placode via chemotaxis mediated by the Sdf1-Cxcr4 chemokine signaling pathway [13]. Similarly, trigeminal precursors are initially widely scattered but then undergo Sdf1/Cxcr4-dependent chemotaxis to converge into a coherent placode [14]. Less is known about the otic and epibranchial placodes, which in zebrafish form in rapid succession from a broad field of contiguous gene expression that includes *pax8*, *pax2a* and *sox3* [15], [16]. The otic domain forms first and induces epibranchial development in more lateral cells [17]. The otic/epibranchial gene expression domain then undergoes marked contraction as the respective placodes coalesce, suggesting active cell migration and convergence. However, there have been no systematic studies of cell migration associated with formation of otic and epibranchial placodes. It is possible that directed cell migration is a general feature common to all placodes, in which case it will be important to identify factors that coordinate these morphogenetic movements.

Directed cell migration often involves navigation along specific ECM domains, attachment to which requires cellular Integrins. Integrins comprise α/β transmembrane heterodimers that bind Fibronectin or Laminin in the ECM to coordinate cell attachment, migration, differentiation and survival [18]–[21]. Integrin-ECM binding triggers several signal transduction pathways, including Ras-MAPK and PI3K signaling, to regulate rapid reorganization of the actin cytoskeleton as well as changes in gene expression. In zebrafish, *integrin-* α*5* (*itga5*) has been shown to regulate a number of early developmental processes, including formation of regular somite boundaries and proper differentiation of cranial neural crest [22]–[24]. Expression is initially widespread, but near the end of gastrulation *itga5* is restricted primarily to preplacodal ectoderm [22]. However, there have been no studies of the role of *itga5* in development of preplacodal ectoderm or its derivatives.

Here we investigate the role of *itga5* in morphogenesis of cranial placodes in zebrafish. Impairment of *itga5* function caused no discernable change in development of anterior placodes, but posterior placodes showed a number of developmental defects resulting in disorganization of trigeminal and epibranchial ganglia and significant reduction in the size of the otic placode/otic vesicle. To examine cell migration patterns, time lapse movies were taken of transgenic embryos expressing *pax2a:GFP* (otic/epibranchial precursors) and *neuroD:EGFP* (trigeminal precursors). Analysis of control (non-morphant) embryos showed that the otic/epibranchial and trigeminal domains normally coalesce by highly focused convergence of cells from within their respective fields. Furthermore, new cells continued to enter the *pax2a:GFP* expression domain from more lateral regions in a process of ongoing recruitment. In *itga5* morphants, cell migration was erratic and unfocused, causing inefficient convergence, redistribution of distal preotic cells into epibranchial regions, and failure of recruitment of new cells. Additionally, cells in the otic/epibranchial domain showed a significantly elevated rate of apoptosis, limiting the increase in epibranchial cells and exacerbating the deficiency of otic cells. Further studies revealed strong genetic interactions between *itga5* and Fgf. For example, the cell death defect was rescued by misexpressing Fgf8. Furthermore, *itga5* morphants showed changes in gene expression that mimic the effects of reducing Fgf signaling; and knockdown of the Fgf-mediator *erm*, which normally causes no overt defects by itself, significantly enhanced the defects in *itga5* morphants. Finally, we showed that proper expression of *itga5* requires *dlx3b*, *dlx4b* and *pax8*, which are regulated by Fgf. These data support a model in which *itga5* coordinates directed cell migration into posterior placodes and augments Fgf signaling to promote cell survival and tissue patterning within the otic/epibranchial domain.

## Results

### *itga5* is required for proper development of posterior placodes

**Figure 1:**
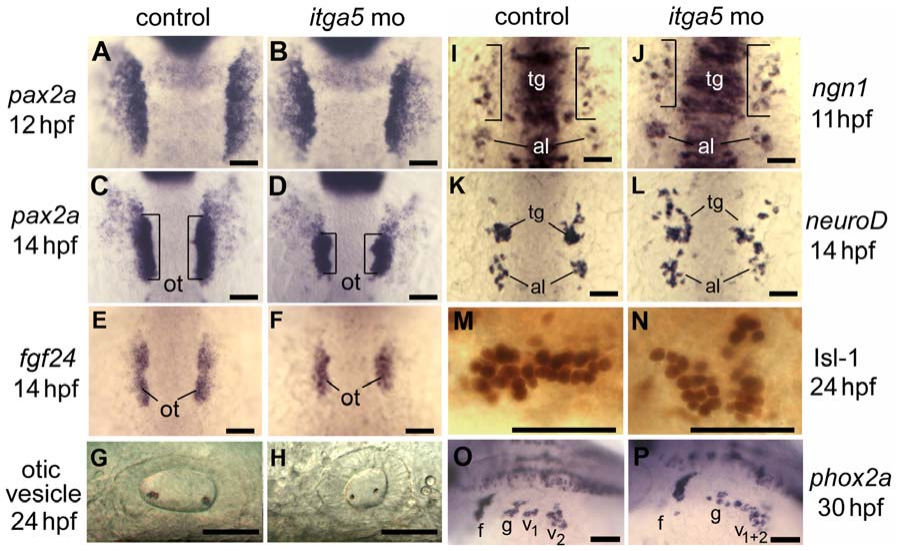
Knockdown of *itga5* impairs morphogenesis of posterior cranial placodes. (**A–D**) *pax2a* expression at 12 and 14 hpf in the otic/epibranchial domain in control embryos (A, C) and *itga5* morphants (B, D). Expression is normal at 12 hpf in *itga5* morphants but the otic placode (o, brackets) is smaller than normal by 14 hpf. (**E, F**) *fgf24* expression at 14 hpf in a control embryo (E) and *itga5* morphant (F). (**G, H**) Otic vesicles at 24hpf in a control embryo (G) and *itga5* morphant (H). (**I, J**) *ngn1* expression at 11 hpf in a control embryo (I) and *itga5* morphant (J). (**K, L**) *neuroD* expression at 14 hpf in a control embryo (K) and *itga5* morphant (L). Precursors of the trigeminal ganglion (tg) and anterior lateral line (al) are indicated. (**M, N**) Anti-Isl-1 immunostaining at 24 hpf in a control embryo (M) and *itga5* morphant (N). (**O, P**) *phox2a* expression in epibranchial ganglia at 30 hpf in a control embryo (O) and *itga5* morphant (P). Facial (f), glossopharyngeal (g), and vagal ganglia (v1+v2) are indicated. A–E, I–K are dorsal views with anterior to the top; G, H, M–P are lateral views with anterior to the left. Scale bar, 50 µm.

*itga5* upregulates in preplacodal ectoderm by 10 hpf [22]. We hypothesized that *itga5* regulates morphogenetic movements associated with formation of discrete cranial placodes. To test this idea, we knocked down *itga5* using morpholinos and monitored subsequent placodal development. We observed no changes in development of anterior placodes, including the anterior pituitary, olfactory, and lens placodes, judging by morphology and early expression of *pitx3*, *cxcr4b* and *foxe3*, respectively (data not shown). In sharp contrast, posterior placodes, including the trigeminal, otic and epibranchial placodes, showed a variety of developmental defects. In control embryos, *pax2a* is strongly expressed at 12 hpf in otic cells, with weaker expression in epibranchial precursors just lateral to the otic domain (Fig. 1A). The otic cells then appear to undergo marked convergence by 14 hpf to form a morphologically visible otic placode (Figs. 1C). Convergence of otic cells is also revealed by expression of the otic marker *fgf24* (Fig. 1E). In *itga5* morphants, the initial otic/epibranchial domain of *pax2a* was normal at 12 hpf. However, the otic placode appeared smaller than normal by 14 hpf (Fig. 1D, F), and the otic vesicle was similarly reduced in size at 24 hpf (compare Fig. 1G, H). Epibranchial ganglia, marked by expression of *phox2a* [25], were highly disorganized in *itga5* morphants, though it is unclear whether the amount of epibranchial tissue was altered (compare Fig. 1O, P). Early development of trigeminal placodes was monitored by expression of *ngn1*, which is initially seen in scattered cells by 11 hpf in control embryos (Fig. 1I). Subsequently, trigeminal cells converge to form compact ganglia by 14 hpf [14], [26], as shown by *neuroD* expression (Fig. 1K). In *itga5* morphants, *ngn1* expression was normal at 11 hpf (Fig. 1J) but *neuroD* staining at 14 hpf revealed that trigeminal cells were still scattered and disorganized (Fig. 1L). Anti-Islet1/2 staining at 24 hpf showed that trigeminal ganglia persisted as disorganized clusters in *itga5* morphants (Fig. 1N). All of the above defects were also observed in *itga5^b926^* mutants [22] (Fig. S1), confirming that the *itga5* morpholino specifically and effectively phenocopies the mutant. In summary, disruption of *itga5* does not alter initial placodal development but impairs assembly of trigeminal, otic and epibranchial placodes after their induction. These defects were relatively specific as gross morphology of the head appeared normal in morphants and mutants.

### Morphogenesis of the otic/epibranchial domain

**Figure 2:**
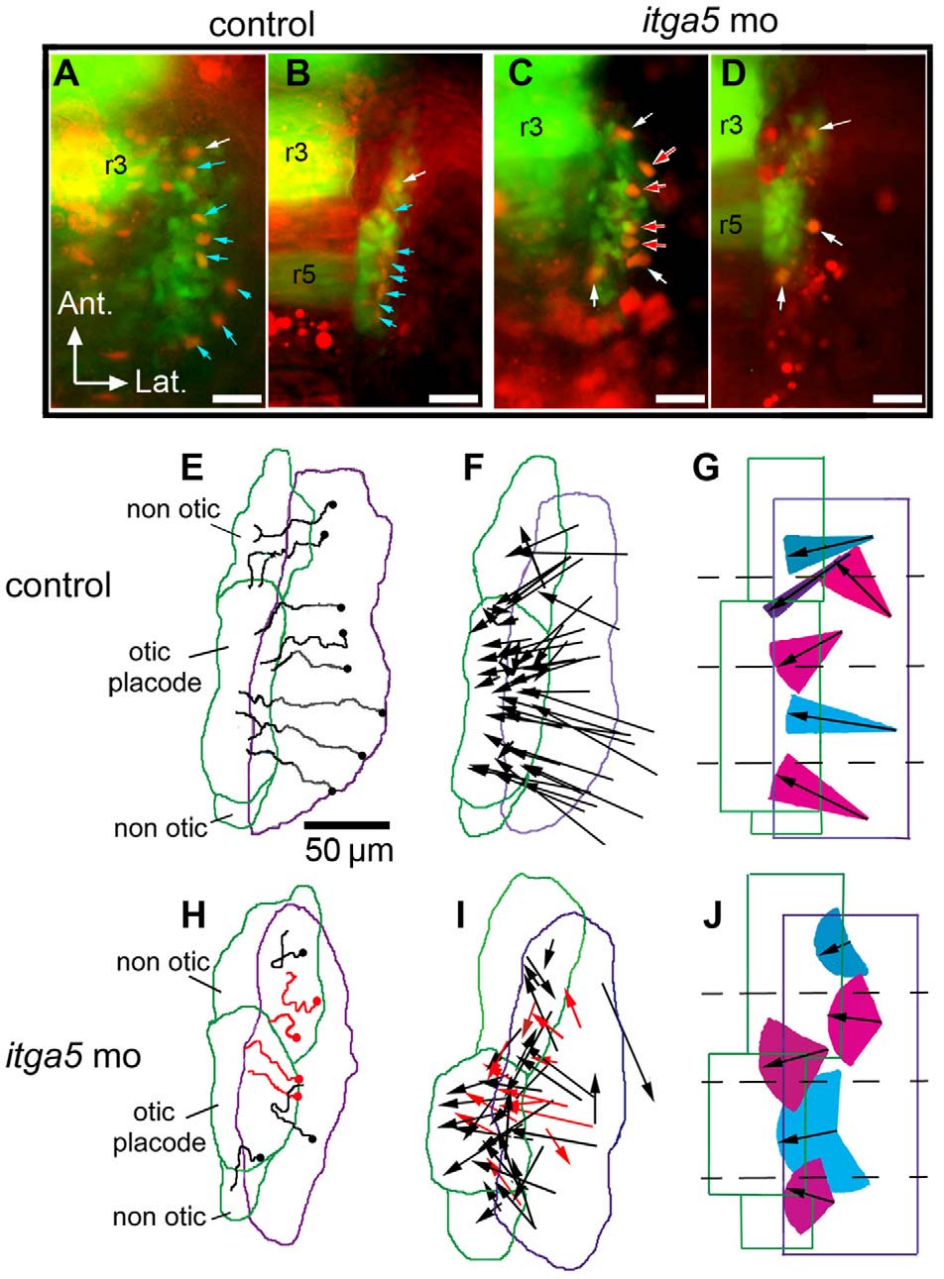
Otic/epibranchial precursors show aberrant migration in *itga5* morphants. (**A–D**) Images from time-lapse movies showing transgenic expression of *pax2a:GFP* (green) and mosaic expression of *cmv:RFP* (red). The first frame (11.5 hpf) and final frame (14.5 hpf) of a control movie (A, B) and *itga5* morphant movie (C, D) are shown. Arrows indicate cells that expressed both GFP and RFP. Blue arrows indicate cells that contributed to the otic domain, and white arrows indicate cells that contributed to non-otic domains. Red arrows indicate cells that lysed during the recording period (C, D). Positions of rhombomeres 3 and 5 (r3, r5) are indicated. (**E, H**) Maps showing the trajectories of all marked cells in the embryos recorded in A–D. Trajectories in red denote cells that lysed during recording (H). The origins of cell trajectories are marked with dots. The initial and final positions of the *pax2a:GFP* domain are indicated by purple and green boundaries, respectively. Final positions of the otic placode and non-otic domains are indicated. (**F, I**) Vector maps showing net displacement of all cells tracked in 5 control movies (F) and 4 *itga5* morphant movies (I). Red arrows indicate cells that died during recording (I). (**G, J**) Summaries of average migration patterns of cells in different quadrants of the *pax2a:GFP* domain in control embryos (G) and *itga5* morphants (J). Arrow length indicates the mean of the net displacement of cells in the indicated region, and colored cones represent the range of angle of net displacement. Quadrants 1 and 2 contained cells contributing to both otic and non-otic domains, which were grouped separately. All images depict the right half of the embryo with lateral to the right and anterior to the top. Scale bar, 50 µm.

We hypothesized that the abnormal development of posterior placodes seen in *itga5* morphants arose in part through defective cell migration. To test this, we tracked cell movements in time-lapse movies using transgenic backgrounds in which precursor cells were labeled with GFP. To monitor otic/epibranchial morphogenesis, we used a *pax2a:GFP* transgenic line that recapitulates the expression of endogenous *pax2a* in the otic/epibranchial domain and midbrain-hindbrain border [27]. Additionally, the *pax2a:GFP* line shows ectopic expression in rhombomeres 3 and 5 of hindbrain, providing a convenient spatial marker to help gauge positions of individual cells. In pilot studies we found it was often difficult to track individual cells for several hours within the multi-layered otic placode. To enhance cell-discrimination, we injected transgenic embryos with plasmid DNA to express RFP mosaically, permitting independent tracking of RFP and GFP in individual cells (see Materials and Methods). Embryos were recorded in time-lapse from 11.5 hpf to14.5 hpf. An example of early and late frames of a movie of a control embryo (Movie S1) is shown in Fig. 2A,B. Tracks of RFP-labeled cells in the same specimen are summarized in Fig. 2E. Most cells migrated in a relatively straight line to contribute to the otic placode, with little deviation in the angle of migration. Similar patterns were documented in a total of five control embryos. The net displacement of all RFP-positive cells (n = 42) tracked in these five embryos is represented in Fig. 2F. To assess typical cell behaviors in different regions, we divided the *pax2a:GFP* domain into quadrants along the anteroposterior axis and calculated the mean net displacement and the range of the angle of displacement for cells in each quadrant (Fig. 2G). Several conclusions regarding patterns of migration emerged from this analysis. First, cells beginning near the medial edge of the *pax2a:GFP* domain usually migrated only short distances to build up this part of the otic placode, whereas cells in more lateral positions migrated much longer distances with a predominant medial vector. Second, in addition to a general medial migration, there was also a marked centripetal convergence into the otic placode from the broader field of GFP-positive cells. That is, many cells in the anterior end of the field showed a posterior trajectory while cells in the posterior showed an anterior trajectory. Third, a few labeled cells in the first and second quadrants did not contribute to the otic placode but instead migrated to the position just anterior of the otic placode. The anterior and posterior non-otic domains of *pax2a:GFP* contribute predominantly to epibranchial ganglia and, based on the overlap between *pax2a:GFP* and *neuroD:EGFP*, the anterior non-otic domain also contains precursors of the anterior lateral line (data not shown). Fourth, we observed numerous cases in which GFP-negative cells neighboring the *pax2a:GFP* domain subsequently entered the domain and became GFP-positive (Fig. S2). Indeed, 29% of tracked cells (12/42 cells in five embryos) exhibited this pattern. Presumably these cells were induced to express *pax2a* once they migrated into range of inductive Fgf signaling, reflecting a process of ongoing recruitment.

Recordings of *itga5* morphants revealed a number of striking differences from control embryos. First, cells in *itga5* morphants showed much more erratic migration patterns, with frequent changes in direction (Fig. 2H–J, Movie S2). Although the total distance traveled was comparable to that seen in control embryos, the meandering course of cell migration in *itga5* morphants resulted in a marked reduction in net displacement (the straight-line distance from start to finish). Accordingly, the mean efficiency of migration (net displacement/total distance) was only .49±.15 in *itga5* morphants compared to .71±.08 in control embryos (Fig. 2I; Table 1). Moreover, the range in angle of net displacement was much greater in all quadrants in *itga5* morphants (Fig. 2J). Second, inefficient cell migration led to aberrant partitioning of the *pax2a:GFP* domain in *itga5* morphants. Specifically, no cells from the first quadrant (0/6), and fewer than half of cells from the second quadrant (6/13), migrated into the otic placode (Fig. 2I,J). This is in contrast to control embryos in which half (3/6) of cells in the first quadrant and 82% (9/11) of cells in the second quadrant converged into the otic placode (Fig. 2F,G). Consequently, *itga5* morphants formed a smaller otic placode and a correspondingly enlarged anterior domain of non-otic cells. Third, we observed no examples of recruitment of neighboring cells into the *pax2a:GFP* domain in *itga5* morphants. That is, all cells tracked in *itga5* morphants (43/43 cells in 4 embryos) were both RFP-positive and GFP-positive throughout the recording period, whereas no neighboring RFP-positive cells were observed to enter the *pax2a:GFP* domain and become GFP-positive. Finally, unlike control embryos, *itga5* morphants showed a striking incidence of cell-lysis within the *pax2a:GFP* domain. For example, 12/43 (27%) of RFP-positive cells tracked in *itga5* morphants lysed during the course of recording (Fig. 2C,D,H,I), whereas none of the 42 cells tracked in control embryos were observed to lyse (Fig. 2F). In contrast, we detected no consistent changes in the rate of cell division in *itga5* morphants compared to controls based on the pattern of BrdU incorporation (Fig. S3). Additionally, tracking of more lateral RFP-positive cells, which never entered the otic-epibranchial domain, revealed that cell migration patterns are relatively normal outside the domain of elevated *itga5* expression (Fig. S4, Table 1). Together these data show that *itga5* is required specifically for normal migration and survival of cells in the otic/epibranchial domain. In the absence of *itga5*, the otic placode is reduced in size by a combination of inefficient convergence, increased cell death, and faulty recruitment of new cells into the *pax2a* domain.

**Table 1:** Knockdown of *itga5* impairs the efficiency of directed cell migration.

Transgenic marker	Experimental condition	Net displacement (µm)±SD	Total distance (µm)±SD	Efficiency of migration (net/total µm)±SD
*pax2a:gfp* (otic/epibranchial)	control *itga5*mo *itga5*mo+*hs:fgf8*	49.4±17.0 32.1±12.5† 28.8±11.3†	68.7±22.5 64.9±24.9† 61.0±8.9†	0.71±0.08 0.49±0.15† 0.46±0.17†
*pax2a:gfp* (otic/epibranchial)	control-mosaic* *itga5*mo-mosaic*	54.9±11.1 28.3±13.3†	68.8±13.4 71.0±19.3	0.79±0.04 0.40±0.17†
*neuroD:gfp* (trigeminal)	control *itga5*mo *itga5*mo+*hs:fgf8*	56.6±9.1 44.4±17.0† 35.9±21.2†	76.8±10.5 97.7±27.3† 61.4±19.4†	0.74±0.06 0.45±0.13† 0.54±0.20†
non-placodal cells	control *itga5*mo	54.7±12.3 40.4±6.2	70.7±14.6 61.3±11.4	0.76±0.02 0.66±0.10

*Indicates genotype of RFP-labeled cells transplanted into *pax2a:gfp* host embryo.

† Significantly different from control value based on t-test.

### Retrospective tracking of epibranchial precursors

**Figure 3:**
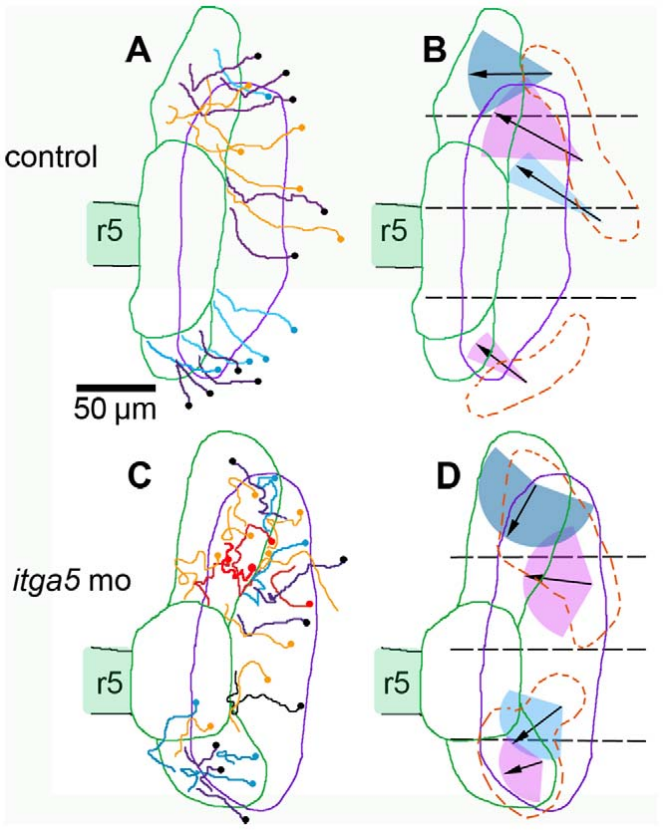
Trajectories of non-otic cells tracked in reverse. (**A, C**) Maps showing the trajectories of GFP positive cells pooled from the time-lapse movies in Fig. 2 of control embryos (A) and *itga5* morphants (C). Trajectories in orange indicate cells originally tracked by coexpression of GFP and RFP. All other trajectories represent cells tracked retrospectively by GFP alone. Dots indicate origins of tracked cells. Trajectories in red denote cells that died during recording (C). (**B, D**) Summaries of average migration patterns on non-otic cells in different quadrants in control embryos (B) and *itga5* morphants (D). The mean length of net displacement (arrows) and range of angle of net displacement (colored cones) are indicated. The dashed orange lines indicate regions from which non-otic cells originated. Initial and final positions of the *pax2a:GFP* domain are represented by the purple and green boundaries, respectively. The position of rhombomere 5 (r5) is indicated. Lateral is to the right and anterior is to the top. Scale bar, 50 µm.

Although the above data provided detailed information about the otic placode, random RFP-labeling marked few cells in the non-otic domains (5/42 in control embryos, Fig. 2F). However, we were able to track additional cells in the non-otic domain back to their origins based solely on GFP expression. Cells tracked in this way showed migration patterns consistent with those previously tracked by RFP-labeling (Fig. 3A). The majority of cells contributing to the anterior non-otic domain converged from the regions adjacent to the first two quadrants whereas all cells in the posterior domain converged from the fourth quadrant (Fig. 3B). Surprisingly, the majority (15/19) of these cells originated from lateral regions outside the domain of contiguous *pax2a:GFP* expression, first appearing as sparsely scattered GFP-positive cells before migrating into the non-otic domains (Fig. 3A,B). Moreover, in keeping with their more lateral origins, these cells were recruited relatively late during the recording period. Whereas recruitment of otic cells usually occurred by 12 hpf (range, 11.7–12.5 hpf), recruitment of non-otic cells occurred around 12.7 hpf (range, 12.5–13.3 hpf) (Fig. S2). This is consistent with our previous findings that otic cells are specified earlier and, through upregulation of *fgf24*, subsequently induce epibranchial precursors from adjacent tissue [17].

In *itga5* morphants, cell migration was highly erratic and the majority (24/27) of non-otic cells originated from within the contiguous domain of *pax2a:GFP* expression (Fig. 3C,D). Additionally, both anterior and posterior non-otic domains were enlarged at the expense of the otic placode. Despite these abnormalities, the general mechanism of sequential induction of epibranchial precursors was found to operate relatively normally in *itga5* morphants based on expression of *fgf24* (Fig. 1F) and *sox3* (see below). Note we did not observe cell lysis amongst retrospectively tracked cells because this technique focused solely on cells that survived until the end of the recording period. Together, these data confirmed that *itga5* is required for normal migration and survival cells contributing to non-otic domains.

### Cell autonomous requirement for *itga5* function

**Figure 4:**
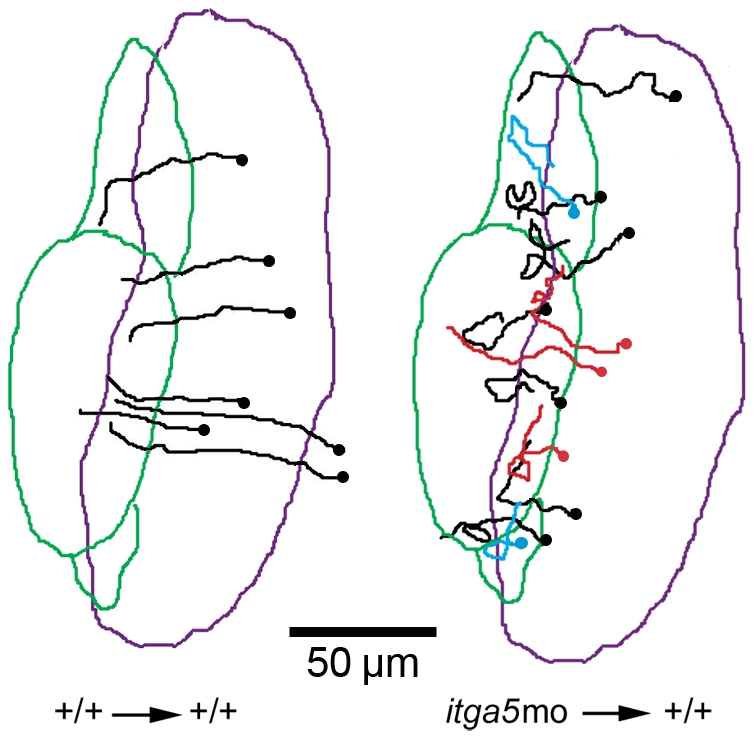
Cell-autonomous requirement for Itga5 in otic/epibranchial cells. (**A**) Trajectories of cells transplanted from a wild type donor into a wild type host and tracked by time-lapse from 11.5–14.5 hpf. Data show tracks of 7 cells from a single embryo. (**B**) Trajectories of cells transplanted from *itga5* morphant donors into wild type host embryos and tracked by time-lapse from 11.5–14.5 hpf. A total of 16 cells from 3 embryos were tracked, though some were not included on the map to avoid confusion. Red tracks represent cells that underwent lysis during the time-lapse. The purple and green boundaries represent the initial and final *pax2a* domain during time-lapse recording. Dots represent the initial positions of cells. Images show dorsal views with anterior to the top. Scale bar, 50 µm.

To examine whether the defect in morphogenesis of otic/epibranchial precursors is a direct effect of *itga5* knockdown, we transplanted *itga5* morphant cells into wild-type embryos and tracked their migration using time-lapse imaging from 11.5–14.5 hpf. As in non-mosaic *itga5* morphants, migration of isolated *itga5*-MO cells in wild-type host embryos was highly erratic (Fig. 4), with a migration efficiency of only 0.44±0.03 (n = 16, Table 1). Additionally, 19% of the tracked cells (n = 3/16) lysed during recording. In contrast, when wild-type cells were transplanted into wild-type hosts, migration efficiency was 0.71±0.08 (n = 7) and no cell lysis was detected. These data show that *itga5* is required cell autonomously for proper migration and survival of cells in the otic/epibranchial domain.

### Morphogenesis of the trigeminal placode

**Figure 5:**
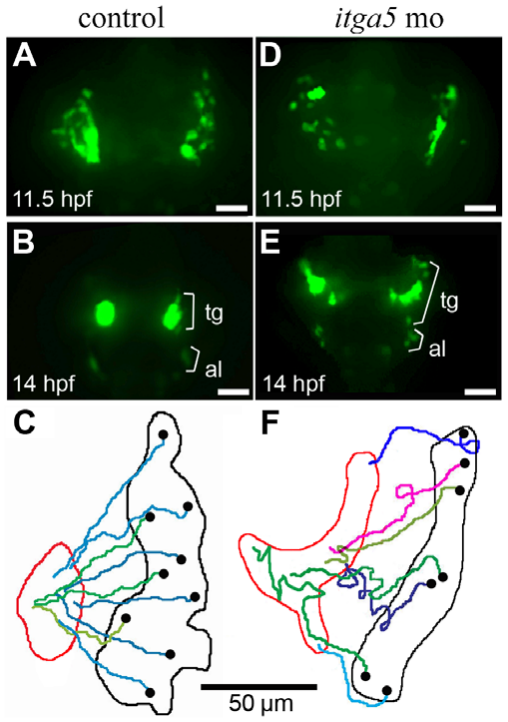
Convergence of trigeminal precursors is impaired in *itga5* morphants. (**A, B, D, E**) Images of time-lapse movies showing transgenic *neuroD:EGFP* expression in the first (11.5 hpf) and final (14 hpf) frames of a control movie (A, B) and *itga5* morphant movie (D, E). Positions of precursors of the trigeminal ganglion (tg) and anterior lateral line (al) are indicated. (**C, F**) Maps showing the trajectories of individual trigeminal precursors in the control embryo (C) and *itga5* morphant (F). Black and red boundaries mark the initial and final distribution, respectively, of *neuroD:EGFP*-positive trigeminal precursors. Black dots represent the initial and final positions, respectively, of individual cells. Images show dorsal views with anterior to the top, and summary figures show the right trigeminal field of each embryo, with lateral to the right. Scale bar, 50 µm.

We next conducted time-lapse analysis of morphogenesis of the trigeminal placode in *neuroD:EGFP* transgenic embryos, which begin to express GFP in trigeminal precursors by 11.5 hpf [28]. Because of the small number and broad distribution of *neuroD:EGFP* cells, we could readily track most trigeminal precursors for the duration of recording and therefore did not require additional RFP plasmid injection. Consistent with endogenous gene expression data (Fig. 1), trigeminal cells in control embryos were initially scattered at 11.5hpf but then rapidly converged into a compact placode by 14 hpf (Fig. 5A–C, Movie S3). Tracks of individual cells were relative straight and showed little deviation (Fig. 5C). In *itga5* morphants, the initial distribution of *neuroD:EGFP* cells was normal at 11.5 hpf but subsequent convergence was severely impaired (Fig. 5D–F, Movie S4). Migration of trigeminal cells was highly erratic with frequent changes in direction (Fig. 5F). Hence the mean efficiency of migration was much smaller than normal (Table 1). Moreover, many cells failed to converge by 14 hpf (Figs. 1J, 5E). Trigeminal cells did eventually converge to form poorly organized clusters by 15.5 hpf (not shown). Thus, *itga5* is required for efficient migration and convergence of trigeminal cells as it is for otic/epibranchial cells. However, we did not observe any cell death in *neuroD:EGFP* expressing trigeminal cells in *itga5* morphants. Thus the requirement for survival appears to be restricted to otic/epibranchial precursors.

### *itga5* regulates cell survival in the otic/epibranchial domain

**Figure 6:**
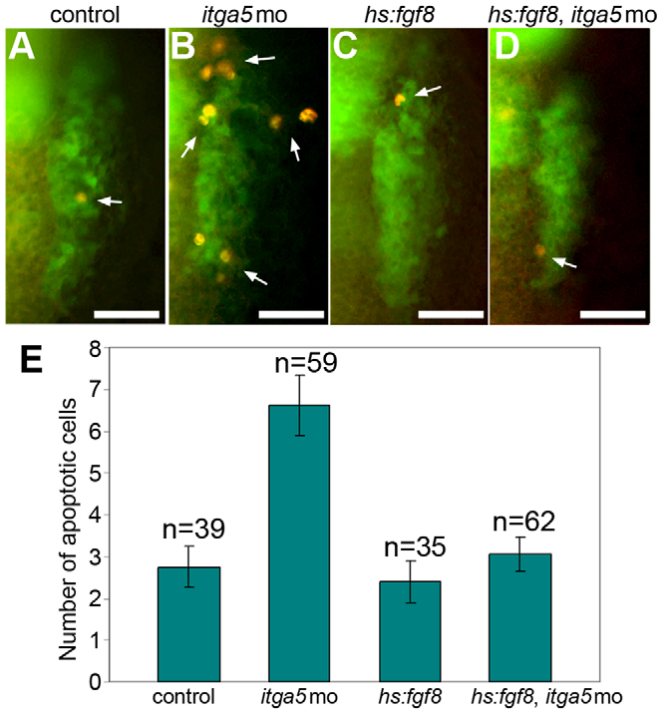
Elevated cell death in *itga5* morphants is rescued by *hs:fgf8*. (**A–D**) Transgenic *pax2a:GFP* embryos immunostained for GFP (green) and Caspase3 (red). Images show dorsal views (anterior up) of the right otic/epibranchial domain in a control embryo (A), *itga5* morphant (B), *hs:fgf8/+* embryo (C) and *hs:fgf8/+* embryo injected with *itga5*-MO (D). White arrows mark apoptotic cells. All embryos were heat shocked at 39°C for 30 minutes beginning at 11.5hpf and fixed at 13.5 hpf. Scale bar, 50 µm. (**E**) Mean number of Caspase3-positive cells in the otic/epibranchial domain in each of the four groups of embryos. Error bars indicate S.E.M.

To confirm whether the cell-lysis observed during time-lapse imaging of otic/epibranchial precursors (Figs. 2,3,4) is due to apoptosis, we visualized apoptotic cells by staining with an antibody directed against activated Caspase-3. This analysis was performed in *pax2a:GFP* transgenic embryos to help visualize the otic/epibranchial region. We observed more than two fold higher levels of apoptosis in *itga5* morphants as compared to control embryos (Fig. 6A,B,E). Elevated apoptosis was limited to the otic/epibranchial domain and did not extend to more anterior ectoderm near the developing trigeminal placodes (data not shown). Similar results were obtained using Acridine Orange (data not shown). We also observed a significant increase in the number of apoptotic cells in *itga5^b926^* mutants (Fig. S1). These data indicate that *itga5* is specifically required for survival of cells in the otic/epibranchial domain.

### *itga5* cooperates with Fgf to regulate otic/epibranchial survival and development

Previous studies have shown that Integrin-ECM binding can promote cell survival through activation of MAPK and PI3K signal transduction. Because otic/epibranchial induction and maintenance requires Fgf signaling, which also operates via MAPK and PI3K activity, we hypothesized that *itga5* augments Fgf signaling to promote cell survival. To test this idea, we used a heat shock-inducible transgene, *hs:fgf8* [29], to elevate Fgf signaling in *itga5* morphants. Activation of *hs:fgf8* expression at 11.5 hpf had no effect on apoptosis in non-morphants (Fig. 6C) but in *itga5* morphants reduced the number of Caspase-3 positive apoptotic cells to normal by 13.5 hpf (Fig. 6D,E). These data support the hypothesis that *itga5* promotes cell survival in part through augmenting Fgf signaling.

We also examined the effects of Fgf misexpression on cell migration in *itga5* morphants. Activation of *hs:fgf8* at 11.5 hpf in *itga5* morphants did not rescue the cell migration defects seen in the otic/epibranchial and trigeminal domains (Fig. S5, Table 1). Thus, elevating Fgf signaling cannot bypass the requirement for *itga5* in regulating coordinated cell migration.

**Figure 7:**
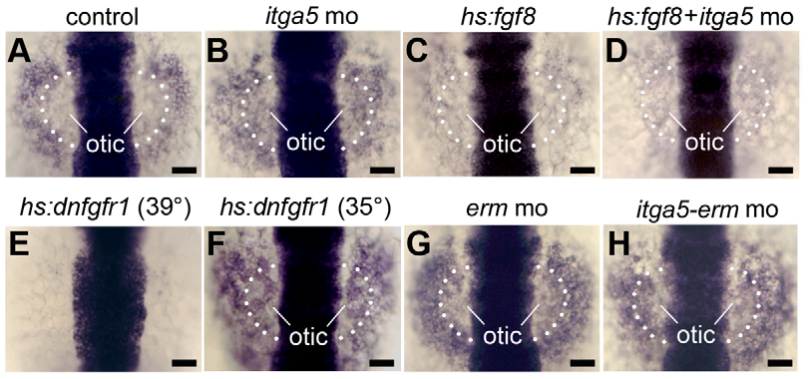
Similar effects of Itga5 and Fgf on *sox3* expression. (**A–H**) *sox3* expression at 12.5 hpf in a control embryo (A), *itga5* morphant (B), *Tg(hs:fgf8/+)* heat shocked at 37°C alone (C) or with *itga5* morpholino (D), *Tg(hs:dnfgfr1/+)* heat shocked at 39°C (E) or 35°C (F), *erm* morphant (G) and *itga5-erm* double morphant (H). The otic region where *sox3* normally downregulates is indicated. Scale bar, 50 µm.

We next tested whether *itga5* influences Fgf-dependent gene expression. Although genes in the Fgf-feedback pathway, including *sprouty4*, *pea3* and *erm* [30]–[32], are often used as indicators for the presence of Fgf, they are not sensitive enough to detect modest reduction in the level of Fgf signaling [33]. However, we recently reported that *sox3* expression provides a sensitive readout of changing levels of Fgf signaling in the otic/epibranchial region, with two distinct threshold responses to Fgf [17]. Specifically, *sox3* is initially induced at a high level in the otic domain in response to moderate Fgf signaling during otic induction [15], [16]. Subsequently, in response to otic expression of *fgf24*, *sox3* expression downregulates to a discrete lower level in the otic domain and is induced at a higher level in abutting epibranchial tissue [17]. We therefore reasoned that if *itga5* morphants experience decreased MAPK or PI3K signaling, expression of *sox3* should remain elevated in the otic domain. In support, *itga5* morphants showed equally heavy expression of *sox3* in the otic and epibranchial domains (Fig. 7B). Similar changes were observed following low level misexpression of dominant-negative Fgf receptor (*hs:dnfgfr1*), while high level activation of *dnfgfr1* ablated *sox3* entirely (Fig. 7E, F). In contrast, activation of *hs:fgf8* resulted in a low level of *sox3* expression throughout the otic-epibranchial domain in both control embryos and *itga5* morphants (Fig. 7C, D). Thus, the effects of *itga5* knockdown on *sox3* expression mimic the effects of modest reduction of Fgf signaling and can be reversed by overexpression of *fgf8*, supporting the notion that *itga5* normally augments Fgf signaling.

Note that the above changes in *sox3* expression do not necessarily reflect changes in cell fate. For example, retention of high levels of *sox3* in the otic domain in *itga5* morphants, and in embryos weakly expressing *dnfgfr1*, does not indicate wholesale switching of otic to epibranchial fate since such embryos still produce substantial otic vesicles. Likewise, retention of elevated *sox3* in the otic domain of *fgf24* morphants does not impair otic development, nor does forced over-expression of *sox3* [17]. Thus, knockdown of *itga5* appears to cause only a modest reduction in cell signaling sufficient to alter *sox3* expression, but not cell fate, within the otic domain.

**Figure 8:**
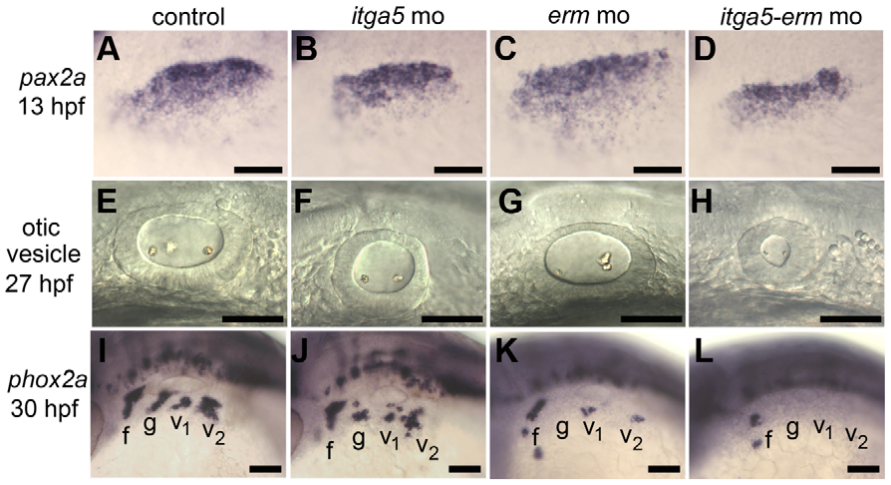
*itga5* and *erm* interact during otic and epibranchial development. (**A–D**) Otic/epibranchial expression of *pax2a* at 13 hpf. **(E–H**) Otic vesicle morphology in at 27 hpf. (**I–L**) *phox2a* expression in epibranchial ganglia at 30 hpf. Positions of facial (f), glossopharyngeal (g) and vagal (v1 and v2) ganglia are indicated. All images show lateral views with dorsal up and anterior to the left. Scale bar, 50 µm.

To further explore the relationship between *itga5* and MAPK signaling, we examined the genetic interaction between *itga5* and *erm*, a direct MAPK-target that helps mediate the effects of Fgf signaling [31], [32], [34]. Because *erm* is partially redundant with *pea3*, knockdown of *erm* alone has negligible effects on gross morphology [34, and our unpublished observations], with the exception of a variable incidence of otolith deficiencies in the otic vesicle (Fig. 8G). In addition, early placodal development appears normal in *erm* morphants (Fig. 8C), though otic expression of *sox3* shows slightly less pronounced downregulation compared to control embryos (Fig. 7G). Thus *erm* morphants provide a sensitized background for detecting further reduction in MAPK signaling. As observed earlier, *itga5* morphants showed a reduced amount of otic tissue (Fig. 8B, F), and simultaneously knocking down *erm* enhanced this deficiency (Fig. 8D, H). While *itga5* morphants produced abundant epibranchial neurons, albeit in a disorganized pattern (Fig. 8J), *erm* morphants showed a marked deficiency of epibranchial ganglia (Fig. 8K) and *erm-itga5* double morphants produced almost none at all (Fig. 8L). Thus, *itga5* and *erm* work together to control otic and epibranchial development, possibly by acting through the same pathway. These data, together with the effects of *itga5*-MO on *sox3* expression and the ability of *hs:fgf8* to rescue the cell death phenotype in *itga5* morphants, support the idea that *itga5* normally augments Fgf signaling to promote cell survival and regulate differential gene expression in otic/epibranchial precursors. In contrast, *itga5* provides a unique function required for directed cell migration that cannot be replaced by elevating Fgf, but which is nevertheless likely to influence how many cells experience Fgf signaling (see Discussion).

### Regulation of *itga5* expression

**Figure 9:**
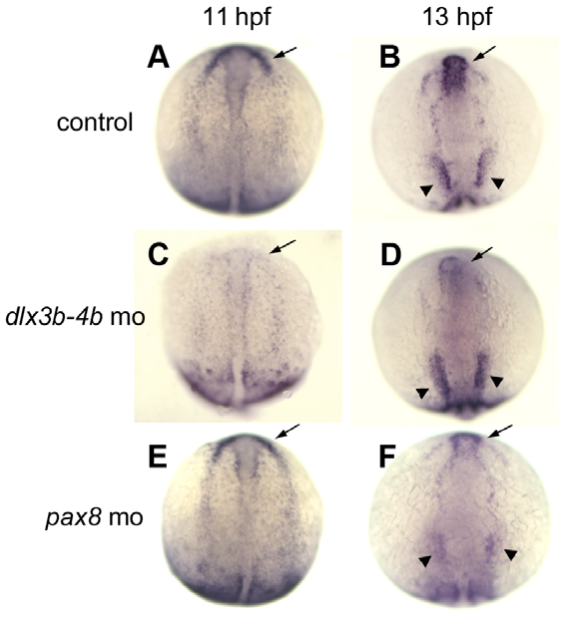
Differential spatial regulation of *itga5* by *dlx*3b/*4b* and *pax8*. (**A–F**) *itga5* expression at 11 hpf and 13 hpf in control embryos (A, B), *dlx3b-dlx4b* double morphants at (C, D), and *pax8* morphants (E, F). Regions where expression normally upregulates in precursors of anterior placodes (arrows) and otic/epibranchial precursors (arrowheads) are indicated. Images show dorsal views with anterior to the top.

To elucidate a more complete picture of the pathway in which *itga5* acts, we sought to identify the upstream regulators of *itga5* expression. Expression of *itga5* shows elevated expression in a horseshoe-shaped pattern marking the preplacodal ectoderm at 10 hpf [22]. By 11 hpf expression strongly upregulates in the anterior-most portion of the preplacodal domain (Fig. 9A), which gives rise to the anterior pituitary, nasal and lens placodes [11]. By 12 hpf expression expands in the otic/epibranchial domain and further intensifies by 13 hpf (Fig. 9B). The spatial pattern of *itga5* expression strongly resembles that of *dlx3b* and *dlx4b*, which are the earliest preplacodal markers in zebrafish and are together required for proper development of many placodal derivatives [7], [35], [36]. We therefore examined whether *dlx3b/4b* genes are required for proper expression of *itga5*. Knockdown of *dlx3b/4b* did not prevent initiation of preplacodal expression of *itga5*, but subsequent upregulation and maintenance of *itga5* in the anterior preplacodal domain were severely impaired (Fig. 9C, D). Surprisingly, however, expression of *itga5* in the otic/epibranchial ectoderm domain was relatively normal in *dlx3b/4b* double morphants (Fig. 9D). Because *pax8* regulates early aspects of otic and epibranchial development [17], [37], [38], we examined expression of *itga5* in *pax8* morphants. Expression of *itga5* was normal in *pax8* morphants through 11 hpf (Fig. 9E) but expression failed to upregulate properly in the otic domain by 13 hpf (Fig. 9F, and data not shown). In contrast, *itga5* expression in the anterior preplacodal domain was relatively normal in *pax8* morphants. In summary, *dlx3b/4b* is required for upregulation and maintenance of *itga5* in anterior preplacodal ectoderm, whereas *pax8* is required for upregulation in the otic/epibranchial domain.

## Discussion

**Figure 10:**
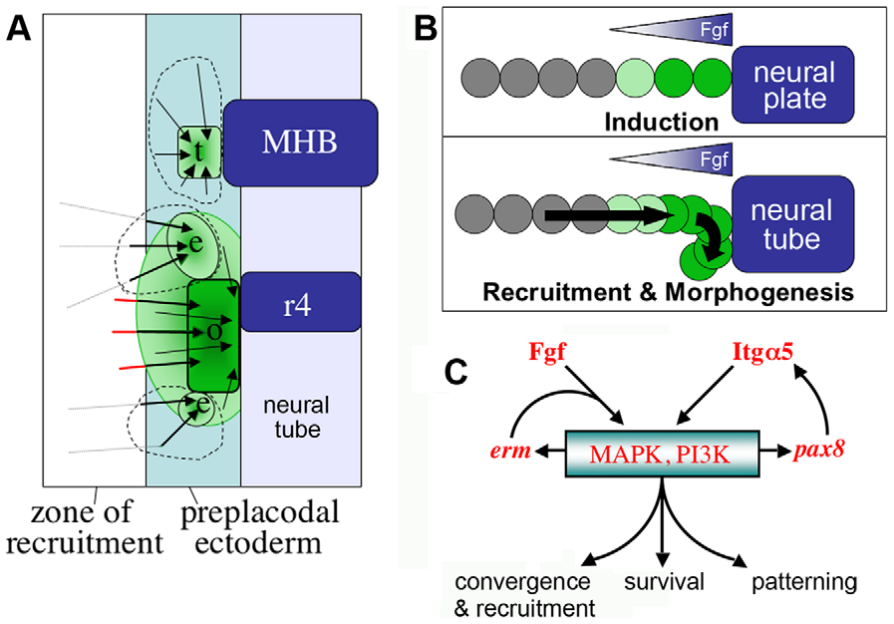
Model for regulation of posterior placode development by *itga5*. (**A**) Summary of cell migration during morphogenesis of trigeminal (t), epibranchial (e) and otic (o) placodes. Arrows indicate migration routes of cells tracked in *neuroD:EGFP* and *pax2a:GFP* expression domains (green). Dashed circles indicate the general areas from which trigeminal and epibranchial precursors were tracked. Most epibranchial precursors were not detected until relatively late (12.5–13.2 hpf) when they first activated *pax2:GFP* in a scattered pattern while still lateral to the contiguous domain of expression. We infer these cells originated from more lateral positions within the zone of recruitment (dashed tracks). RFP-positive otic cells were observed to migrate into the *pax2a:GFP* domain from nearby in the zone of recruitment (red tracks) between 11.7–12.5 hpf. Positions of the midbrain-hindbrain border (MHB) and rhombomere 4 (r4) are indicated. (**B**) A model for recruitment of otic/epibranchial cells. An initial otic domain (green) is induced by dorsally expressed Fgfs. Subsequently, *itga5*-dependent medial migration drives convergence of the otic field and draws new cells into range of inductive signaling. (**C**) Model for *itga5* in reinforcing Fgf signaling. Erm helps mediate Fgf signaling, which begets more *erm* expression. Fgf also activates *pax8*, which stimulates upregulation of *itga5* in the otic/epibranchial domain, further reinforcing Fgf signaling. Fgf acts primarily through the MAPK and PI3K pathways, and Itga5 facilitates these pathways through a variety of mechanisms (see text for details). Together these functional interactions control directed cell migration, cell survival, and gene expression within the otic/epibranchial field.

We have shown an essential role for *itga5* in proper formation of trigeminal, otic and epibranchial placodes. Cell motion analysis in GFP-transgenic backgrounds shows that cells normally converge from broad fields into compact placodes through highly directed migration. Overall, cells contributing to these diverse placodes originate from distinct regions with only limited overlap (Fig. 10A). The data further show that the otic and epibranchial domains continue to expand after gastrulation through ongoing recruitment of cells from more lateral nonneural ectoderm (Fig. 10B). In *itga5* morphants, cell migration is much less directional: Though the total distance traveled is comparable to normal, cells frequently change direction and backtrack, causing significant impairment of convergence and complete abrogation of recruitment. Loss of *itga5* also impairs patterning and cell survival in the otic/epibranchial domain. Several lines of evidence indicate that *itga5* interacts with Fgf signaling to regulate this region. First, misexpression of Fgf8 is sufficient to rescue the cell death phenotype in *itga5* morphants (Fig. 6). Second, *itga5* morphants show changes in *sox3* expression that mimic the effects of reduced Fgf signaling, and this effect can be reversed by misexpressing *fgf8* (Fig. 7). Third, knockdown of *erm* enhances the patterning defects in *itga5* morphants (Fig. 8). Finally, proper upregulation of *itga5* requires the Fgf-target gene *pax8* (Fig. 9), suggesting that *itga5* acts in a feed-forward loop to reinforce Fgf signaling in the otic/epibranchial domain. Together, our data support a model in which *itga5* coordinates cell migration, differentiation and survival in posterior cranial placodes, in part by enhancing Fgf signaling (Fig. 10C).

### Role of Integrin in directed cell migration

Preplacodal ectoderm forms by the end of gastrulation and then quickly breaks into discrete placodes through directed cell migration [9]–[14]. Integrins are good candidates for regulating such morphogenetic processes, but there have been no previous studies of the role of Integrins in placodal development in any species. We chose to study *itga5* in zebrafish because it is initially expressed at a low level throughout the preplacodal ectoderm and later upregulates in various placodal derivatives as they form [22] (Fig. 9). Surprisingly, placodes derived from the anterior portion of the preplacodal ectoderm (anterior pituitary, olfactory and lens placodes) appear to develop normally in *itga5* morphants. In contrast, *itga5* knockdown impairs directed migration of precursors in posterior placodes, resulting in disorganization of trigeminal and epibranchial ganglia and significant deficiency of otic tissue. Interestingly, cells were observed to migrate long distances in *itga5* morphants albeit in a non-directed manner, indicating that Itga5 does not simply provide traction on the migratory substrate. Instead, Itga5 could help provide directionality through sensing adhesive gradients in the ECM (haptotaxis). Fibronectin-1 (Fn1) is an ECM component that strongly binds Itga5 and is expressed maximally by mesoderm just beneath the neural-nonneural border [39], [40], possibly providing a cue for medial migration. Additionally, Integrin-ECM binding appears necessary for restricting pseudopod production to the leading edge of migratory cells [21]. Otherwise, cells tend to extend multiple pseudopods in random directions, resulting in frequent changes in direction similar to what we have observed in *itga5* morphants. Dynamic regulation of Integrins helps to stabilize pseudopods at the leading edge during chemotaxis of many cell types [18] and plays a vital role in modulating the response of T-lymphocytes to chemokines [41]. The chemokine Sdf1 is required for focal migration of trigeminal precursors [14], and knockdown of *itga5* causes a phenotype similar to loss of the Sdf1 receptor Cxcr4. It is not known whether specific chemokines regulate convergence of otic or epibranchial cells. Otic placode cells express the chemokine Cxcl14 (Scyba) [42], though knockdown of this gene causes no discernable defects (our unpublished observations). Epibranchial-specific chemokines are yet to be identified.

### Role of Integrin in cell signaling

Itga5 facilitates signaling associated with specification and survival of otic/epibranchial precursors, probably through multiple distinct mechanisms. First, *itga5*-dependent migration draws lateral cells into range of inductive Fgf signaling, thereby promoting recruitment of additional cells into these placodes (Fig. 10B). We showed previously that the number of *pax2a*-positive cells roughly doubles between 11 hpf and 14 hpf, and a similar fold-increase is seen in mutants blocked in cell division, leaving recruitment as the only means available to increase cell number [43]. Second, Integrin-ECM binding is required in many cell types for proper nuclear import of phosphorylated MAPK/ERK [44]–[46]. Otherwise, ERK can continue to be phosphorylated yet accumulate ineffectually in the cytosol. Third, *itga5* could specifically amplify Fgf signaling through direct physical interactions. In mammalian vascular endothelial cells, for example, Integrin-αvβ3 binds Fgf1 or Fgf2 directly to form a ternary complex with FGFR1 and promotes receptor clustering [47]–[49]. Such binding interactions are required for sustained MAPK/ERK signaling. Itga5 can also stimulate PI3K [50], another signal transducer shared by the Fgf pathway that regulates cell migration and fate specification in a variety of cell types [51]–[53].

In the case of otic/epibranchial development, *itga5* is not required for sustained Fgf signaling but appears necessary for achieving a full level of Fgf signaling. Although knockdown of *itga5* does not detectably alter expression of genes in the Fgf synexpression group, such as *erm*, *pea3* or *sprouty4*, such genes are not suitable for detecting modest reduction in Fgf signaling [17], [33]. Fortunately, *sox3* expression shows two distinct threshold-responses to Fgf signaling: Moderate Fgf signaling induces a high level of *sox3* expression in the epibranchial domain whereas elevated Fgf signaling downregulates *sox3* expression to a discrete lower level in the otic domain [17]. The finding that *sox3* expression is inappropriately maintained at a high level in the otic placode of *itga5* morphants (Fig. 7B) provides evidence that Fgf signaling is indeed diminished in this domain. Furthermore, reduced signaling in *itga5* morphants explains the elevated cell death seen throughout the otic/epibranchial domain, since activating *hs:fgf8* rescues this phenotype (Fig. 5). Nevertheless, it is interesting that *sox3* shows normal upregulation in the peripheral/epibranchial domain in *itga5* morphants (Fig. 7B). This process is Fgf-dependent and indicates that moderate Fgf signaling remains sufficient at the edge of the signaling range to properly regulate gene expression in the absence of *itga5* function. Thus, the effect of signaling on patterning can be separated to some extent from regulation of cell survival, with the latter being more sensitive to slight changes in signaling activity.

The link between cell fate specification and cell migration in the otic/epibranchial field is complex. We showed previously that otic and epibranchial fates are specified sequentially: The otic placode forms first and expresses *fgf24*, which then induces epibranchial development through upregulation of *sox3* expression in more lateral ectoderm [17]. Based on cell-tracking in the *pax2a:GFP* domain, recruitment of otic and epibranchial cells occurs at correspondingly different times. Recruitment of otic cells occurs mostly between 11.5–12 hpf, whereas the majority of epibranchial cells are recruited between 12.5–13.3 hpf from a slightly more lateral domain. Given the 40-minute lag between onset of transcription to accumulation of GFP, these times agree well with dynamic changes in *sox3* expression in otic vs. epibranchial precursors [15]–[17]. Changes in these processes in *itga5* morphants provide some insight as to how morphogenesis and fate specification are coordinated. Because recruitment of new cells fails almost entirely in *itga5* morphants, fates are specified within a fixed population of *pax2a:GFP*-positive cells. Inefficient cell migration appears to contribute to a marked deficiency of otic cells and corresponding increase in non-otic domains. It is understandable that slow migration impedes peripheral cells from entering the otic domain in a timely manner, but why don't peripheral cells intercalate with otic cells at later times? There is possibly a timing mechanism that excludes new cells from the otic domain after some critical period. The transition is possibly related to how long cells are exposed to varying Fgf concentrations, which could co-regulate fate-specification with activation of genes that mediate contact-dependent repulsion. Further studies are needed to test these ideas.

## Materials and Methods

### Strains

Control embryos were derived from the AB line (Eugene, OR). In various experiments we used transgenic lines *Tg(pax2a:GFP)^e1^* [27], *Tg(neuroD:EGFP)^nl1^* [28] and *Tg(hsp70:fgf8)^x17^* [29], and the mutant line *itga5^b926^* [22].

### In-situ hybrization and immunostaining

In-situ hybridization was performed as described in [54]. Primary antibodies were used for Isl-1 (Hybridoma Bank, 1∶100), Caspase3 (R&D Systems, 1∶100) and GFP (Santa Cruz Biotechnology, 1∶250). Secondary antibodies were HRP-conjugated anti-mouse (Vector Labs, 1∶200) and Alexa-conjugated anti-mouse and anti-rabbit (Invitrogen, 1∶50).

### Morpholino injections

Morpholino sequences for *itga5*, *dlx3b*, *dlx4b* and *pax8* have been previously described and tested [23], [36], [38]. For *erm* knockdown we used *erm*-MO 5′-CTTGCTGGTCATAAAACCCATCCAT-3′, which is nearly identical to *etv5/erm-*MO described previously [34]. Embryos were injected at the one-cell stage with 5 ng each of the indicated MOs.

### RFP misexpression

CMV∶RFP plasmid was injected into one-cell stage embryos at a concentration of 75 ng/ul to give mosaic expression of RFP.

### Time lapse imaging

Embryos were mounted on glass slides in drops of 0.8% low-gel agarose surrounded by plastic frame. After mounting, embryos were hydrated and coverslips were affixed with petroleum jelly. Embryos were imaged for time-lapse using a Zeiss Axioimager2-ApoTome. Fluorescent images were taken every 3 minutes using Axiovision 4.6.3 software.

### Cell tracking and vector maps

To correct for inadvertent movement of the embryo during recording, GFP expression in rhombomeres 3 and 5 was used to provide a fixed reference in each frame. ImageJ software was used to manually track individual cells. Tracks were then projected onto representational maps using Photoshop. Calculations for displacement, total distance and the angle for each vector were calculated using ImageJ software.

### Generating chimeric embryos

*Tg(CMV∶RFP)* transgenic embryos were coinjected with 100 ng/nl *CMV∶RFP* plasmid DNA and 5 ng/nl of *itga5* morpholino or just plasmid DNA and used as donors. At the 1000-cell stage donor cells were transplanted with a glass needle into *pax2a:GFP* transgenic host embryos. Chimeras containing cells in the otic/epibranchial domain were selected for time-lapse imaging, as described above.

## Supporting Information

Figure S1 **Abnormal development of posterior placodes in** ***itga5^b926/b926^*** **mutants.** (**A, B**) *pax2a* expression at 14 hpf in the otic/epibranchial domain in a wild-type embryo (A) and *itga5* mutant (B). Otic placodes (o, brackets) are indicated. (**C, D**) *neuroD* expression at 14 hpf in a control embryo (C) and *itga5* morphant (D). Precursors of the trigeminal ganglion (tg) and anterior lateral line (al) are indicated. (**E, F**) Immunolocalization of Pax2 (green) and Caspase 3 (red) in a wild-type embryo (E) and *itga5* mutant (F). (**G, H**) Otic vesicles at 24hpf in a wild-type embryo (G) and *itga5* mutant (H). (**I, J**) *phox2a* expression in epibranchial ganglia at 30 hpf in a wild-type embryo (I) and *itga5* mutant (J). Facial (f), glossopharyngeal (g), and vagal ganglia (v1+v2) are indicated. A–E are dorsal views with anterior to the top; G–J are lateral views with anterior to the left. Scale bar, 50 µm.
(TIF)

Figure S2 **Recruitment of lateral cells into the** ***pax2a:GFP*** **domain.** Representative frames from a movie of a *pax2a:GFP* transgenic embryo injected with *cmv:RFP* plasmid DNA. RFP-positive cells originating from a position lateral to the otic/epibranchial domain were tracked as they entered the *pax2a:GFP* domain and activated expression of GFP. White arrows indicate the positions of two cells with respect to domains of GFP alone or both GFP and RFP. A map of the migration patterns of the two tracked cells is indicated, with the purple and green borders marking the initial and final positions, respectively, of the *pax2a:GFP* domain.
(TIF)

Figure S3 **Knockdown of** ***itga5*** **does not alter proliferation.** Embryos were incubated in BrdU beginning at 11.5 hpf, fixed at 13.5 hpf and immunostained for BrdU (green) and Pax2a (red).
(TIF)

Figure S4 **Knockdown of** ***itga5*** **does not perturb cell migration in more lateral regions.** Maps of migration patterns of RFP-positive cells observed in movies of a *pax2a:GFP/+* control embryo and a *pax2a:GFP/+* embryo injected with *itga5-*MO. Embryos were injected with *cmv:RFP* plasmid at the one-cell stage and imaged from 11.5 hpf to 14.5 hpf. In the *itga5* morphant, lateral cells migrated normally for most of the filming period. Two cells showed deviations only after nearing the otic/epibranchial domain where *itga5* expression normally upregulates. Nevertheless, migration efficiency was not significantly different in *itga5* morphants relative to controls (p = 0.10) (see Table 1).
(TIF)

Figure S5 **Elevating Fgf does not rescue the cell migration defect in** ***itga5*** **morphants.** Maps of cell trajectories in *pax2a:GFP/+*; *hs:fgf8/+* and *neuroD:GFP/+*; *hs:fgf8/+* embryos injected with *itga5*-MO. To assist in cell-tracking, embryos were also injected at the one-cell stage with *cmv:GFP*. Embryos were heat shocked at 11 hpf and filmed from 11.5–14.5 hpf. Cells were tracked by RFP expression in the *pax2a:GFP* background, or by GFP expression in the *neuroD:GFP* background. Initial boundaries of transgenic GFP expression are shown in purple and black, whereas final boundaries are shown in green and red.
(TIF)

Movie S1 **Migration of cells in a** ***pax2a:GFP*** **control embryo.** In addition to GFP expression, the embryo was injected at the one-cell stage with *cmv:RFP* plasmid DNA, providing mosaic expression to facilitate cell-discrimination within the otic placode. The movie runs from 11.5 hpf to 14.5 hpf.
(MOV)

Movie S2 **Migration of cells in a** ***pax2a:GFP*** **embryo injected with** ***itga5*** **-MO.** In addition to GFP expression, the embryo was injected at the one-cell stage with cmv:RFP plasmid DNA, providing mosaic expression to facilitate cell-discrimination within the otic placode. The movie runs from 11.5 hpf to 14.5 hpf.
(MOV)

Movie S3 **Migration of cells in a** ***neuroD;EGFP*** **control embryo.** The movie runs from 11.5 to 14.0 hpf.
(MOV)

Movie S4 **Migration of cells in a** ***neuroD;EGFP*** **embryo injected with** ***itga5*** **-MO.** The movie runs from 11.5 to 14.0 hpf.
(MOV)
