# Supplementary figures and images for: Integrin-α5 Coordinates Assembly of Posterior Cranial Placodes in Zebrafish and Enhances Fgf-Dependent Regulation of Otic/Epibranchial Cells
Source: PLoS One. 2011 Dec 2;6(12):e27778. doi: 10.1371/journal.pone.0027778 (PMC3229493; doi:10.1371/journal.pone.0027778)

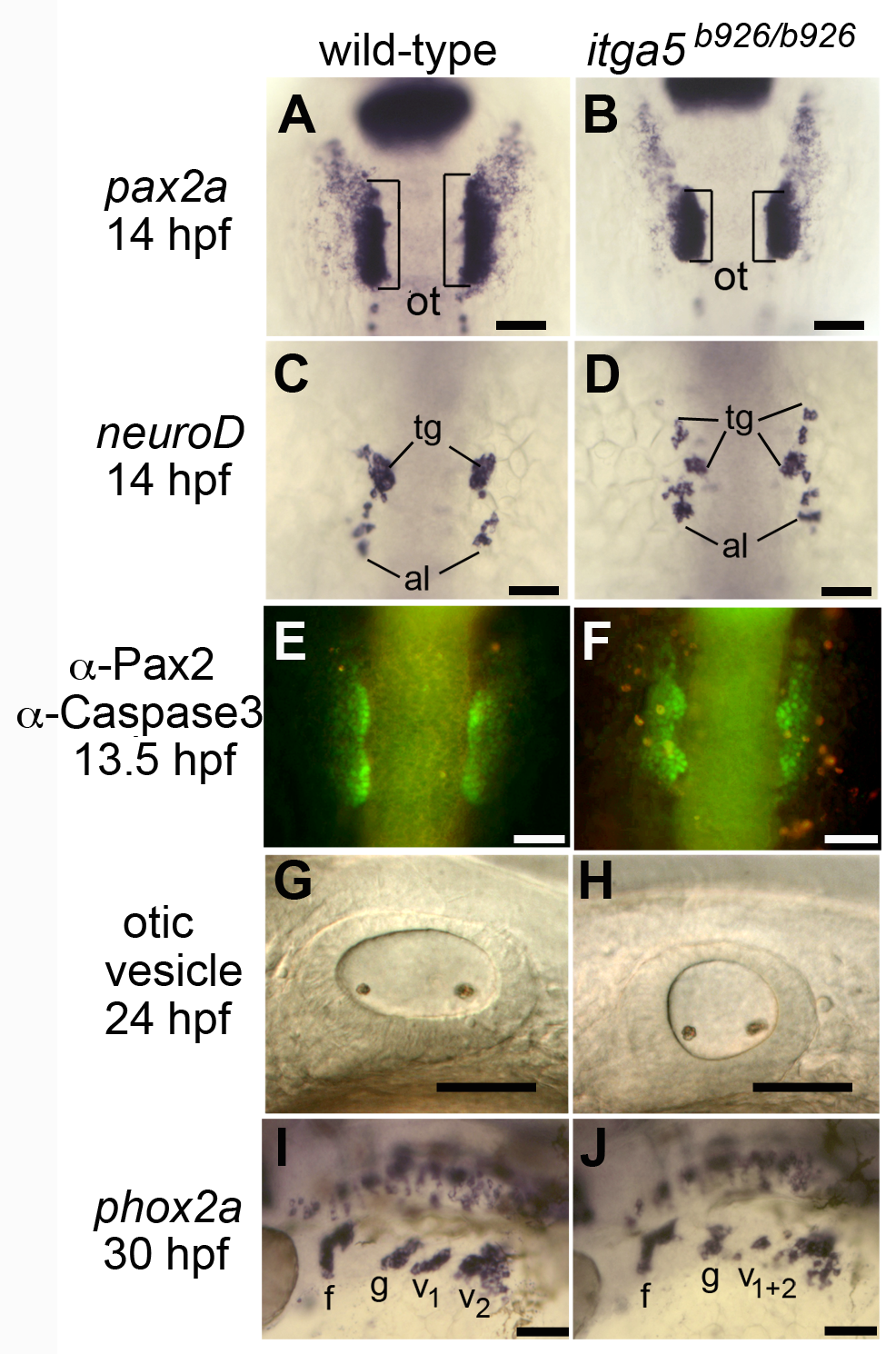

Supplement: Figure S1 — Abnormal development of posterior placodes in itga5b926/b926 mutants. (A, B) pax2a expression at 14 hpf in the otic/epibranchial domain in a wild-type embryo (A) and itga5 mutant (B). Otic placodes (o, brackets) are indicated. (C, D) neuroD expression at 14 hpf in a control embryo (C) and itga5 morphant (D). Precursors of the trigeminal ganglion (tg) and anterior lateral line (al) are indicated. (E, F) Immunolocalization of Pax2 (green) and Caspase 3 (red) in a wild-type embryo (E) and itga5 mutant (F). (G, H) Otic vesicles at 24hpf in a wild-type embryo (G) and itga5 mutant (H). (I, J) phox2a expression in epibranchial ganglia at 30 hpf in a wild-type embryo (I) and itga5 mutant (J). Facial (f), glossopharyngeal (g), and vagal ganglia (v1+v2) are indicated. A–E are dorsal views with anterior to the top; G–J are lateral views with anterior to the left. Scale bar, 50 µm. (TIF) [file pone.0027778.s001.tif]

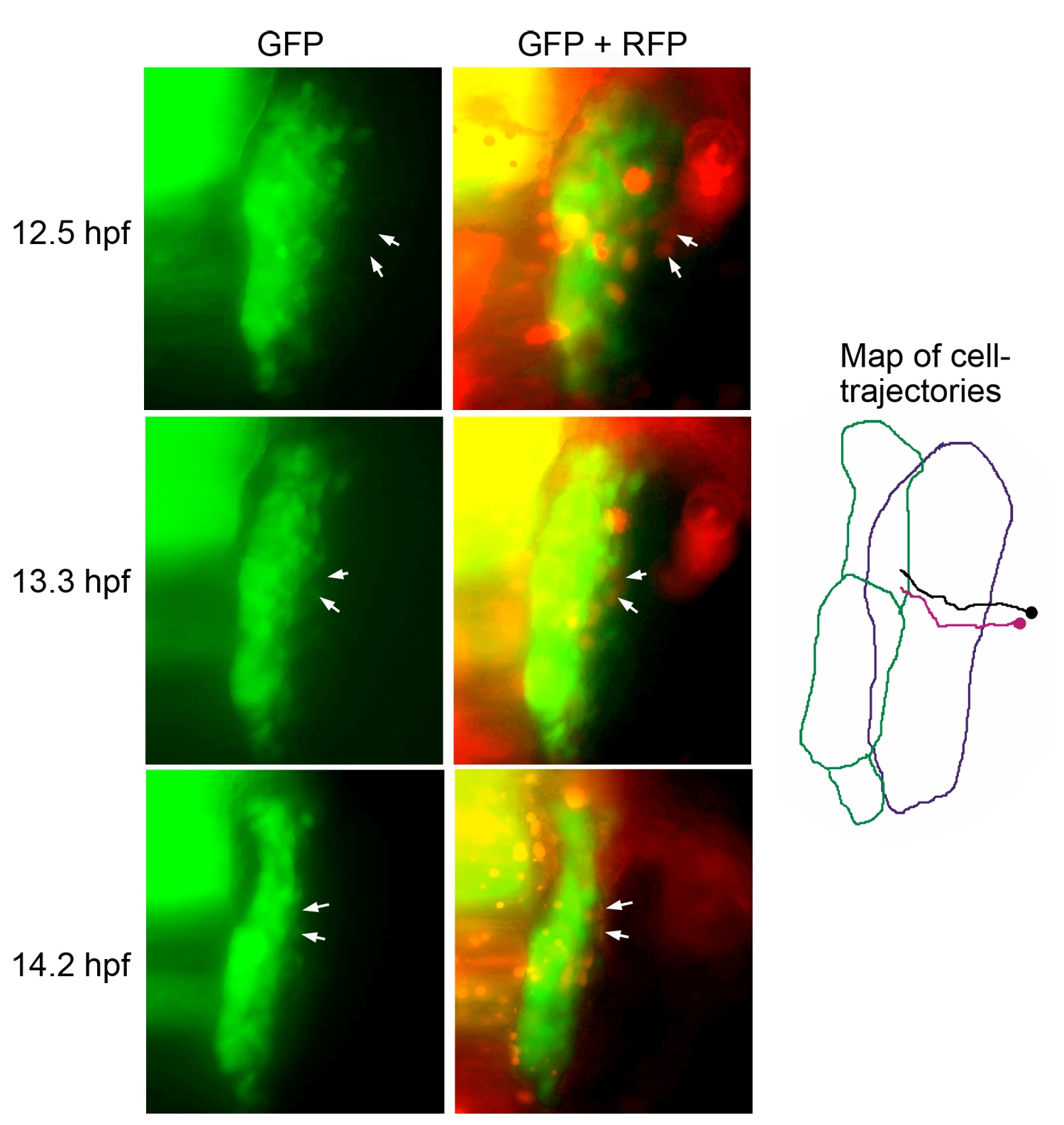

Supplement: Figure S2 — Recruitment of lateral cells into the pax2a:GFP domain. Representative frames from a movie of a pax2a:GFP transgenic embryo injected with cmv:RFP plasmid DNA. RFP-positive cells originating from a position lateral to the otic/epibranchial domain were tracked as they entered the pax2a:GFP domain and activated expression of GFP. White arrows indicate the positions of two cells with respect to domains of GFP alone or both GFP and RFP. A map of the migration patterns of the two tracked cells is indicated, with the purple and green borders marking the initial and final positions, respectively, of the pax2a:GFP domain. (TIF) [file pone.0027778.s002.tif]

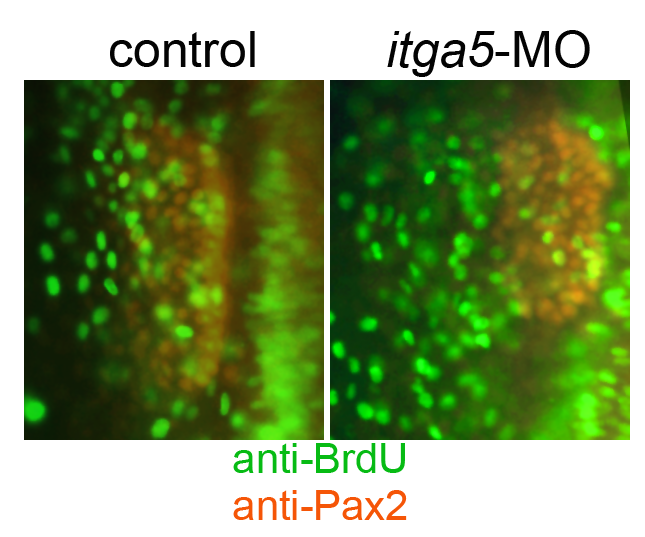

Supplement: Figure S3 — Knockdown of itga5 does not alter proliferation. Embryos were incubated in BrdU beginning at 11.5 hpf, fixed at 13.5 hpf and immunostained for BrdU (green) and Pax2a (red). (TIF) [file pone.0027778.s003.tif]

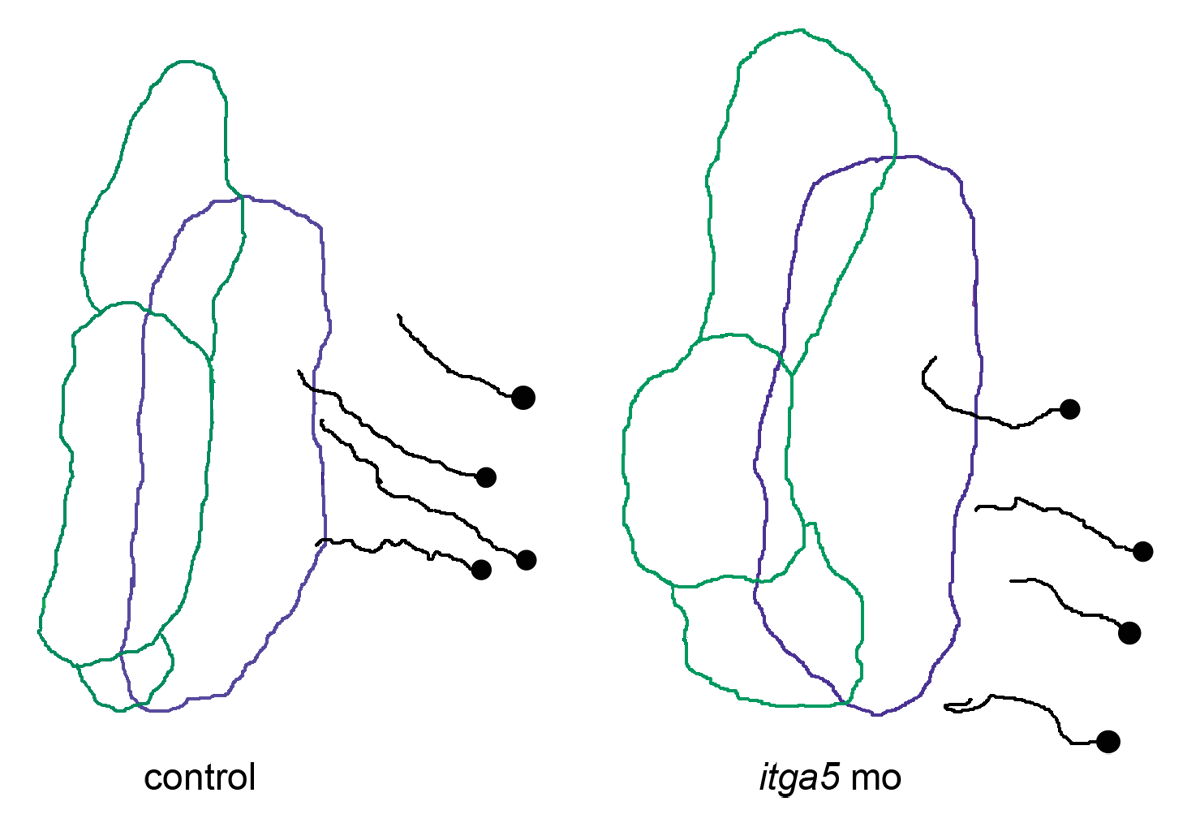

Supplement: Figure S4 — Knockdown of itga5 does not perturb cell migration in more lateral regions. Maps of migration patterns of RFP-positive cells observed in movies of a pax2a:GFP/+ control embryo and a pax2a:GFP/+ embryo injected with itga5-MO. Embryos were injected with cmv:RFP plasmid at the one-cell stage and imaged from 11.5 hpf to 14.5 hpf. In the itga5 morphant, lateral cells migrated normally for most of the filming period. Two cells showed deviations only after nearing the otic/epibranchial domain where itga5 expression normally upregulates. Nevertheless, migration efficiency was not significantly different in itga5 morphants relative to controls (p = 0.10) (see Table 1). (TIF) [file pone.0027778.s004.tif]

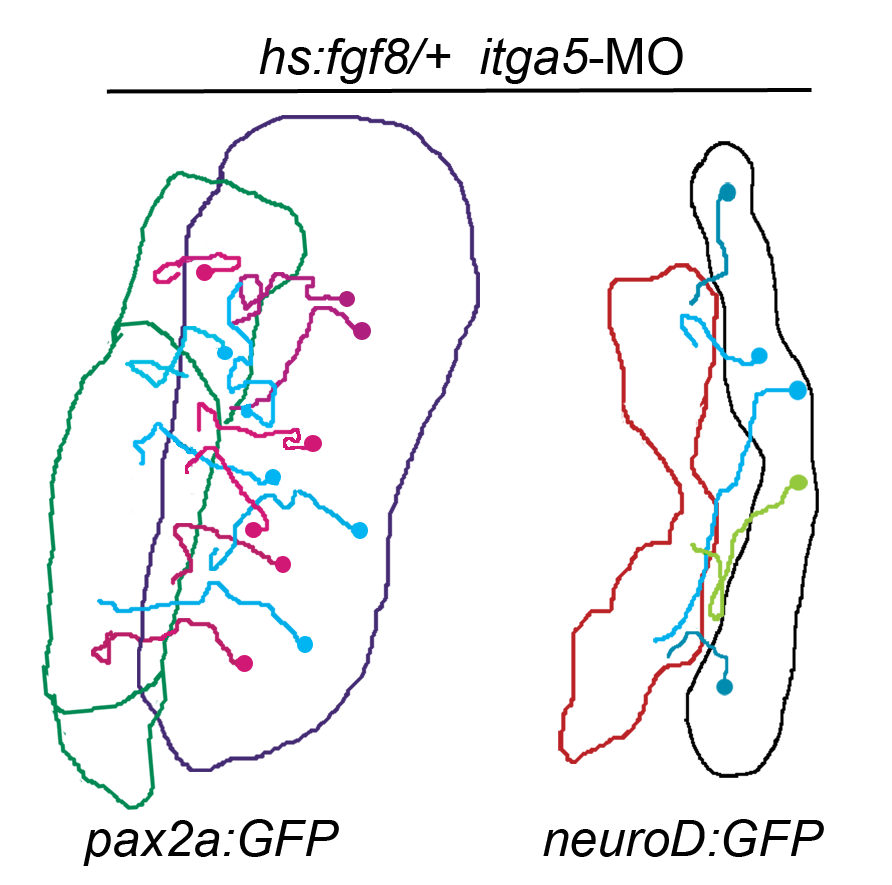

Supplement: Figure S5 — Elevating Fgf does not rescue the cell migration defect in itga5 morphants. Maps of cell trajectories in pax2a:GFP/+; hs:fgf8/+ and neuroD:GFP/+; hs:fgf8/+ embryos injected with itga5-MO. To assist in cell-tracking, embryos were also injected at the one-cell stage with cmv:GFP. Embryos were heat shocked at 11 hpf and filmed from 11.5–14.5 hpf. Cells were tracked by RFP expression in the pax2a:GFP background, or by GFP expression in the neuroD:GFP background. Initial boundaries of transgenic GFP expression are shown in purple and black, whereas final boundaries are shown in green and red. (TIF) [file pone.0027778.s005.tif]
